# Machine Learning Driven Profiling of Cerebrospinal Fluid Core Biomarkers in Alzheimer’s Disease and Other Neurological Disorders

**DOI:** 10.3389/fnins.2021.647783

**Published:** 2021-03-31

**Authors:** Giovanni Bellomo, Antonio Indaco, Davide Chiasserini, Emanuela Maderna, Federico Paolini Paoletti, Lorenzo Gaetani, Silvia Paciotti, Maya Petricciuolo, Fabrizio Tagliavini, Giorgio Giaccone, Lucilla Parnetti, Giuseppe Di Fede

**Affiliations:** ^1^Laboratory of Clinical Neurochemistry, Section of Neurology, Department of Medicine and Surgery, University of Perugia, Perugia, Italy; ^2^Neurology 5/Neuropathology Unit, Fondazione IRCCS Istituto Neurologico C. Besta, Milan, Italy; ^3^Section of Biochemistry, Department of Medicine and Surgery, University of Perugia, Perugia, Italy; ^4^Section of Neurology, Department of Medicine and Surgery, University of Perugia, Perugia, Italy

**Keywords:** Alzheimer’s disease, biomarkers, dementia, cerebrospinal fluid, amyloid-beta, tau, machine learning, clustering analysis

## Abstract

Amyloid-beta (Aβ) 42/40 ratio, tau phosphorylated at threonine-181 (p-tau), and total-tau (t-tau) are considered core biomarkers for the diagnosis of Alzheimer’s disease (AD). The use of fully automated biomarker assays has been shown to reduce the intra- and inter-laboratory variability, which is a critical factor when defining cut-off values. The calculation of cut-off values is often influenced by the composition of AD and control groups. Indeed, the clinically defined AD group may include patients affected by other forms of dementia, while the control group is often very heterogeneous due to the inclusion of subjects diagnosed with other neurological diseases (OND). In this context, unsupervised machine learning approaches may overcome these issues providing unbiased cut-off values and data-driven patient stratification according to the sole distribution of biomarkers. In this work, we took advantage of the reproducibility of automated determination of the CSF core AD biomarkers to compare two large cohorts of patients diagnosed with different neurological disorders and enrolled in two centers with established expertise in AD biomarkers. We applied an unsupervised Gaussian mixture model clustering algorithm and found that our large series of patients could be classified in six clusters according to their CSF biomarker profile, some presenting a typical AD-like profile and some a non-AD profile. By considering the frequencies of clinically defined OND and AD subjects in clusters, we subsequently computed cluster-based cut-off values for Aβ42/Aβ40, p-tau, and t-tau. This approach promises to be useful for large-scale biomarker studies aimed at providing efficient biochemical phenotyping of neurological diseases.

## Introduction

Alzheimer’s disease (AD) is the most common neurodegenerative disorder evolving to dementia (Goedert and Spillantini, 2006). Increasing knowledge of the molecular mechanisms underlying the pathogenesis of AD has progressively improved the protocols employed for its diagnosis in clinical practice (Jack et al., 2018). However, AD is also recognized as a heterogeneous disorder that may occur under several distinct phenotypes which can mimic other forms of dementias and other neurodegenerative conditions (Di Fede et al., 2018). Such phenotypic heterogeneity sometimes makes the differential diagnosis between AD and other similar neurological diseases problematic (Sawyer et al., 2017; de Souza et al., 2019; Villain and Dubois, 2019). Cerebrospinal fluid (CSF) core biomarkers for AD – i.e., amyloid-beta (Aβ) 42/40 ratio, tau phosphorylated at threonine-181 (p-tau), and total-tau (t-tau) – are largely used in clinical settings, research, and drug trials (Paterson et al., 2018; Gaetani et al., 2020). However, their clinical utility to differentiate AD from non-AD neurodegenerative dementias, such as dementia with Lewy bodies (DLB) or frontotemporal dementia (FTD), is less established (Bartlett et al., 2012; Molinuevo et al., 2014). For a long time, manual enzyme-linked immunosorbent assay (ELISA) has been widely employed as the reference method for the analysis of the CSF AD biomarkers. However, its broad-scale use is critically hampered by the assay variability, which influences the measurement of the analytes and the interpretation of the outcome data, especially in the routine clinical context (Mattsson et al., 2012; Le Bastard et al., 2015). Due to these concerns, ELISA was recently replaced in worldwide laboratories by fully automated assays – such as chemiluminescence enzyme immunoassay (CLEIA) – which offer grounds to cut sample manipulation steps and to reduce the intra- and inter-laboratory variability for CSF biomarker measurement (Kollhoff et al., 2018). Nevertheless, there is still a need for harmonization of CSF biomarker assays across centers involved in AD diagnostics (Mattsson-Carlgren et al., 2020). For instance, the lack of established universal biomarker cut-offs makes the calculation of internal reference values mandatory for each laboratory both for clinical and research purposes. This calculation is often critically influenced by the choice and composition of AD and control groups; the clinically defined AD group may include patients affected by other forms of dementia (e.g., FTD and DLB) due to misdiagnosis, while the control group is often very heterogeneous due to the inclusion of subjects diagnosed with other neurological diseases (OND) who underwent lumbar puncture (LP) for diagnostic purposes. On the one hand, OND may better represent the real cases afferent to neurology clinics compared to healthy subjects. However, the heterogeneity of the inclusion criteria adopted in each center for the definition of OND controls represents a source of variability for the calculation of biomarkers cut-off values. In addition, the absence of standardized methodological and statistical approaches represents one of the most critical issues to study the distribution of CSF core AD biomarkers in different subgroups of patients and to validate in larger cohorts the cut-off values able to discriminate between AD and other AD-mimicking disorders (Simrén et al., 2020). In this context, unsupervised machine learning approaches may overcome both misdiagnosis and the lack of standardization of inclusion criteria providing unbiased cut-off values and data-driven patient stratification according to the sole distribution of biomarkers. In this work, we took advantage of the reproducibility of automated determination of the CSF core AD biomarkers to compare two large cohorts of patients diagnosed with different neurological disorders and enrolled in two centers with established expertise in AD biomarkers. We applied an unsupervised Gaussian mixture model (GMM) clustering algorithm and found that our large series of patients could be classified in six clusters according to their CSF biomarker profile, some presenting a typical AD-like profile and some a typical non-AD profile. By considering the frequencies of clinically defined OND and AD subjects in clusters, we subsequently computed cluster-based cut-off values for Aβ42/Aβ40, p-tau, and t-tau.

## Materials and Methods

### Patients

A total of 616 prospectively collected CSF samples from patients referring to the Neurology Clinic, University of Perugia (cohort 1), and from the Carlo Besta Neurological Institute, Milan (cohort 2), were used in this study. All patients underwent a standardized assessment including medical history, physical and neurological examination, laboratory tests, neuropsychological evaluation, and brain imaging (computed tomography or magnetic resonance imaging, MRI). 18Fluoro-2-deoxyglucose positron emission tomography (FDG-PET), dopamine transporter single photon emission computed tomography (DaT-Scan), and electroencephalogram were also performed in selected cases, according to clinical suspicion. According to the purposes of our investigation, clinical diagnoses were made by consensus in a multidisciplinary meeting of neurologists with a deep expertise in the field of neurodegenerative diseases, without knowledge of CSF results. Therefore, we did not consider the most updated criteria for AD diagnosis, based on A/T/(N) classification (Jack et al., 2018), but rather we defined patients as affected by AD or other neurological disorders only according to the available clinical criteria, as follows. Patients with neurodegenerative disorders included 257 patients with probable AD (Dubois et al., 2007) both at dementia and prodromal (MCI) stages, 50 frontotemporal dementia (FTD) patients (Faber, 1999), 56 patients with Parkinson’s disease (PD) (Postuma et al., 2015), 7 PD with dementia (PDD) patients (Emre et al., 2007), 21 patients with dementia with Lewy bodies (DLB) (McKeith et al., 2017), 58 patients with atypical parkinsonism or parkinsonism of different etiology (Gilman et al., 2008; Armstrong et al., 2013; Höglinger et al., 2017; Rektor et al., 2018), 1 patients with amyotrophic lateral sclerosis (ALS) (Traynor et al., 2000), 8 patients with Creutzfeldt-Jakob disease (CJD) (Manix et al., 2015), 27 patients with normal pressure hydrocephalus (NPH) (Relkin et al., 2005), and 2 patients with genetically confirmed degenerative spinocerebellar ataxia (SCA). Patients were classified as having subjective cognitive decline (SCD) if they complained cognitive deficits but neuropsychological evaluation was normal or showed subtle deficits not fulfilling criteria for mild cognitive impairment (MCI) (8 patients). Patients with stable MCI (sMCI) showed unchanged neuropsychological results after 1-year follow-up (20 patients). Other diagnostic groups included vascular dementia (5 patients) (Román et al., 1993), cerebral amyloid angiopathy (CAA) (Smith and Greenberg, 2003) (3 patients), autoimmune encephalitis (8 patients) (Graus et al., 2016), encephalopathies of different etiology (2 patients), relapsing-remitting multiple sclerosis (MS) (Thompson et al., 2018) (6 patients), and cognitively impaired late-onset epilepsy (1 patient) (Scheffer et al., 2017). Patients categorized as having cerebrovascular diseases (CVD) showed significant brain small vessel disease at MRI (i.e., white matter changes, microbleeds, and lacunar infarcts) without fulfilling diagnostic criteria for VaD and CAA (3 patients). Dementia of unknown origin (uDEM) was defined for those subjects in which brain imaging including both MRI and nuclear imaging excluded vascular and neurodegenerative origins (3 patients). Cognitively unimpaired patients referring to our centers for psychiatric disorders or neurological conditions like headaches, seizures, mononeuropathies, and polyneuropathies, in which brain imaging did not reveal gross abnormalities nor underlying neurodegenerative diseases, were classified as control subjects with other neurological diseases (OND) (71 patients).

### Samples Collection and Analysis

Lumbar puncture was performed according to international guidelines (Teunissen et al., 2009); 10–12 mL of CSF was collected in sterile polypropylene tubes (Sarstedt^®^ tubes, code: 62.610.210) and centrifuged for 10 min (2000 × *g*), at room temperature. Aliquots of 0.5 mL were frozen at −80°C in polypropylene tubes (Sarstedt^®^ tubes, code: 72.730.007). CSF samples were analyzed on the fully automated chemiluminescent platform Lumipulse G600-II (Fujirebio Inc) for β-amyloid 1-42 (Aβ42), β-amyloid 1-40 (Aβ40), t-tau and p-tau (Thr181) levels. For cohort 1, all the CSF samples were analyzed directly in their 0.5 mL storage tubes, while for cohort 2 samples were analyzed by transferring them in Hitachi^®^ polystyrene sample caps (code: 80351). Throughout this work, Aβ42/Aβ40 ratio was used since it represents a more robust marker of amyloidosis with respect to the sole Aβ42 (Biscetti et al., 2019). Moreover, the use of Aβ42/Aβ40 can also partially compensate the above-mentioned methodological difference between the two centers, since the Aβ absorption due to tube transfer (Toombs et al., 2014) is thought to act similarly for the 1-40 and 1-42 Aβ isoforms (Lewczuk et al., 2006).

### Cohorts Merging

A preliminary experiment was carried out to assess the inter-center variability of the CSF biomarkers and the possibility to merge the two cohorts. A total of 40 CSF samples (20 from each center) were measured with Lumipulse-G automated platforms in the two laboratories by using kits originating from the same batches. The composition of this validation cohort is reported in Supplementary Table 1. The concordance between the measurements was assessed by correlation analysis.

### Statistical Methods

The data analysis was performed by using R software v 3.6 (R Core Team, 2013).

#### Correlation Analysis

Because of the known non-optimal normality of biomarker data (Bellomo et al., 2020a), Passing Bablok regressions (Passing and Bablok, 1983) were preferred to parametric least squares regressions. Confidence intervals (CI) for the fitted parameters were calculated with the bootstrap method (Carpenter and Bithell, 2000). Principal component analysis (PCA) was then applied to the whole dataset to graphically show the absence of a significant separation among samples belonging to different cohorts. The R-package *mcr* was used for these calculations [mcr package | R Documentation (2021)].

#### Cluster Analysis

The GMM algorithm (Figueiredo and Jain, 2002) of the machine learning Python package Scikit-learn v 0.23.2 (Pedregosa et al., 2011) was used for the cluster analysis. Biomarker values were all z-scored prior to the analysis. The optimal number of clusters was chosen minimizing the Bayesian information criterion (BIC) function (Schwarz, 1978). After the clustering, all the samples biomarker values were back-transformed into the original dimensions. Median biomarker values together with the 95% data range were calculated for each cluster. The prevalences of diagnostic categories in each cluster were represented in percentages in a heatmap. Both diagnostic groups and clusters were grouped according to a hierarchical clustering (Rokach and Maimon, 2005; Gu, 2021). Euclidean distance and average linkage were used as parameters for clustering.

#### Calculation of Cut-Off Values

Cut-off values were calculated for OND vs. AD and among clusters by maximizing Youden’s index with the p-ROC package in R v3.6 (Robin et al., 2011). Cut-off CI were calculated by using 2000 bootstrap replicates.

#### Calculation of Cut-Off Values on Age- and Gender-Matched Subsets

Age histogram matching was performed by random exclusion of subjects within bins of 5 years width. Exclusion of samples according to gender was subsequently conducted until *p*-values > 0.25 were obtained by logistic regression (Dobson and Barnett, 2018; glm function | R Documentation, 2021) both for age and gender. Recalculation of cut-off values for the age- and gender-matched subsets was performed as described in section “Calculation of Cut-Off Values.”

## Results

### Patients Demographical Data

A total of 616 patients whose CSF samples were tested for AD biomarker by Lumipulse-G, were included in the study regardless of age and clinical diagnoses. Among them, 257 were clinically diagnosed as AD and 71 cognitively unimpaired subjects affected by minor neurological non-neurodegenerative disorders were classified as OND. The whole cohort originated by merging two sub-cohorts: 303 subjects referred to the biobank of the Neurology clinic of the University of Perugia (cohort 1), while 313 referred to the biobank of the Carlo Besta Neurological Institute of Milan (cohort 2). The demographical details of the subjects included are reported in Supplementary Table 3.

### Merging of the Two Cohorts

**FIGURE 1:**
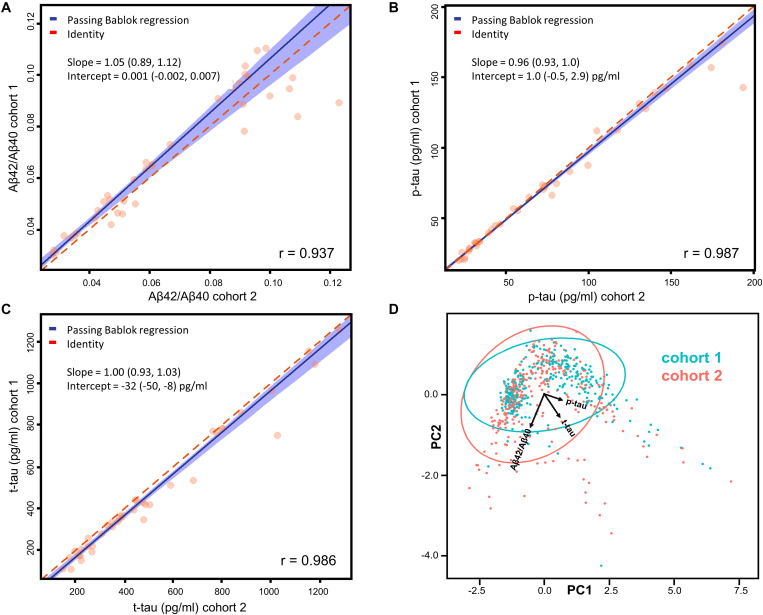
**(A–C)** Passing-Bablok regression analyses of Aβ42/Aβ40, p-tau, and t-tau measured on 40 samples (20 from each cohort) in the two centers. Correlations have been calculated in terms of Pearson’s correlation coefficients (r). Fitted slopes and intercepts with their 95% CI are also shown. **(D)** Plot (PC1 vs. PC2) relative to the PCA performed on the whole dataset with samples belonging to different cohorts highlighted in different colors. The ellipses relative to the 95% data range of each cohort are also shown together with the projections of Aβ42/Aβ40, p-tau and t-tau in the PC1-PC2 space.

Assessment of CSF AD biomarkers with Lumipulse-G CLEIA technology showed very low inter-center variability in external quality control programs (Leitão et al., 2019; Paciotti et al., 2019). However, we performed a small scale inter-center variability study, measuring a total of 40 samples across the two centers involved, to assess the possibility to merge the cohorts from Perugia (cohort 1, 303 subjects) and Milan (cohort 2, 313 subjects). Patients’ diagnoses, mean biomarker values and inter-assay/inter-laboratory coefficients of variations (CV) are reported for all of these samples in Supplementary Table 1. As expected, the mean inter-assay CV of Aβ42/Aβ40 (7%) was lower compared to the ones of Aβ42 (12%) and Aβ40 (9%). Mean inter-assay CV of p-tau (4%) and t-tau (9%) were also relatively low. Correlation and Passing-Bablok linear regression analyses showed a good agreement between the measurements performed in the two centers (Figures 1A–C). A Pearson’s correlation coefficient above 0.9 was found for each of the tested biomarkers and all the measured slopes were equal to 1 within their 95% CI. Intercepts for Aβ42/Aβ40 and p-tau were null within their 95% CI, whereas a non-null negative intercept was obtained for t-tau, although being small compared to usual nominal t-tau values. The results of Passing-Bablok linear regression analysis for Aβ42 and Aβ40 are shown in Supplementary Table 2. As expected (Lewczuk et al., 2006), we found a greater deviance from identity between the measurements of these two peptides performed in the two laboratories with respect to their ratio. We subsequently analyzed all the 616 samples included in the study by means of PCA. Projection of the data into the principal components space (Figure 1D) showed that the measurement from the two centers did not show any significant grouping related to the site of analysis, as shown by the ellipses representative of the 95% data range of the two cohorts.

Both the regression and the PCA analysis showed that the inter-center variability was negligible for Aβ42/Aβ40, p-tau and t-tau. Cut-off values for Aβ42, Aβ42/Aβ40, p-tau and t-tau for cohort 1, cohort 2 and for the two cohorts merged are also reported in the Supplementary Table 4. The calculated cut-off values did not significantly differ within their 95% CI between cohort 1 and 2. Thus, we proceeded to merge the two cohorts for subsequent analyses, without applying any correction factor for Aβ42/Aβ40, p-tau and t-tau.

### Clustering

**FIGURE 2:**
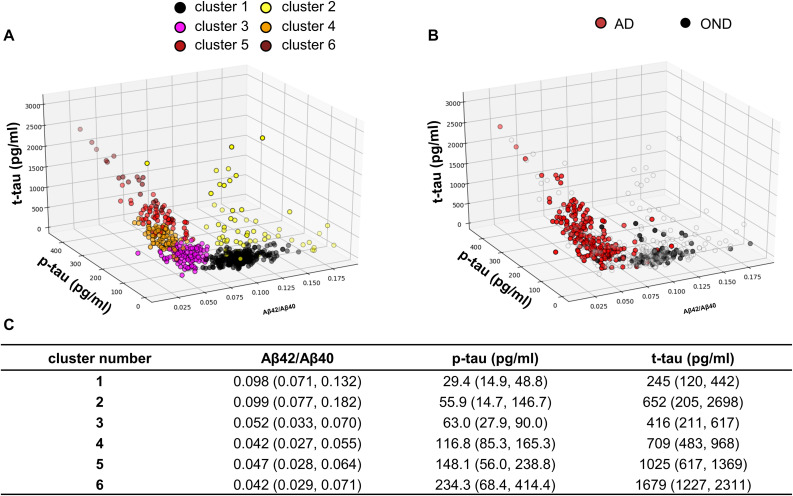
**(A)** Samples distribution in the core AD biomarkers space. The colors indicate the cluster to which the sample is belonging, after GMM analysis. **(B)** Samples belonging to AD patients and OND are highlighted in red and black, respectively. **(C)** Median biomarker values with the 95% data range of each cluster represented in brackets.

For the unsupervised cluster analysis, we included all the subjects who consecutively underwent LP in the two centers and whose CSF was assayed for Aβ42/Aβ40, p-tau and t-tau with Lumipulse-G (*N* = 616). Considering the results of the PCA plotted in Figure 1D, we decided to apply GMM as clustering algorithm (Pedregosa et al., 2011). Other clustering algorithms such as K-means are known for not providing good fittings for anisotropic data. In order to standardize the dimensions of the three biomarkers considered, biomarker values were substituted with the corresponding Z-scores before the analysis and then back-transformed for plotting and data interpretation. The optimal number of clusters was decided according to BIC (Schwarz, 1978). A plot relative to the BIC function is shown in Supplementary Figure 1. Accordingly, the optimal number of clusters turned out to be 6. The results of the unsupervised clustering analysis are shown in Figure 2A, GMM centroids and covariance matrices are reported in Supplementary Table 5. As it can be seen by comparing the 3D scatter plots in Figures 2A,B, most AD and OND samples were assigned to different clusters. In particular, most OND were included in cluster 1 while most AD subjects (95%) were comprised in clusters 3, 4, 5, and 6. Considering the biomarker 95% data ranges of each cluster (Figure 2C), clusters 3–6 corresponded to low Aβ42/Aβ40 values and high values of p-tau and t-tau, which, according to the A/T/(N) criteria (Jack et al., 2018), correspond to the presence of amyloidosis, tauopathy and tau-related neurodegeneration, respectively. These four clusters mainly differed in p-tau and t-tau values (Figure 2C). Among AD patients, for all the AD clusters, the prevalences of demented subjects (*N* = 253) did not significantly differ from the prevalences of subjects in the MCI phase (*N* = 53) by applying Fisher’s exact test for count data. Cluster 1 was instead characterized by higher values of Aβ42/Aβ40 and small values of p-tau and t-tau. Biomarker values in cluster 2 were instead highly variable with respect to the other clusters (wide 95% data ranges for all the three biomarkers). This cluster was characterized by smaller Aβ42/Aβ40 median values with respect to clusters 3–6 and higher p-tau and t-tau median values compared to cluster 1.

**FIGURE 3:**
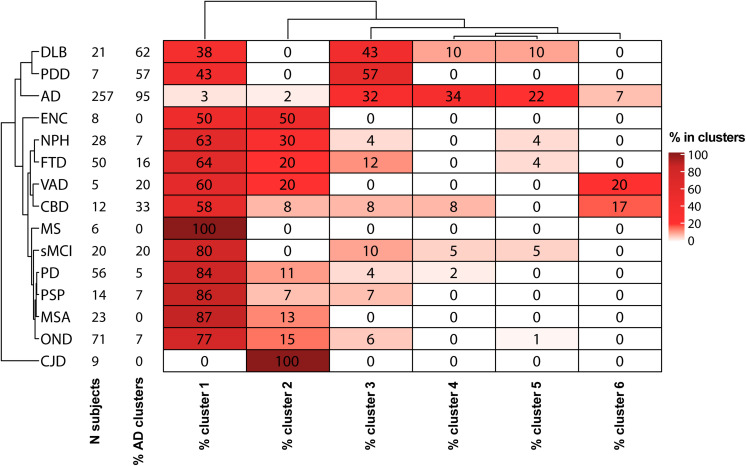
Heatmap descriptive of the GMM cluster analysis results. For each diagnostic category with a sample size (*N* subjects) ≥ 5, the percentages of samples in each cluster are shown. Hierarchical clustering was used for ordering diagnostic groups and clusters.

The percentages of AD patients, OND and other sufficiently represented (N > 6) clinical conditions in each cluster are reported in Figure 3. Interestingly, not only AD but also PDD, DLB, and CBD had a relevant presence (>20%) in clusters 3–6. Conversely, MS, PSP, sMCI, and synucleinopathies without dementia (PD and MSA) majorly colocalize with OND in cluster 1. The composition of cluster 2 was instead highly variable, consisting in the totality of CJD subjects and relevant percentages (≥20%) of NPH, ENC, FTD, and VAD. Considering the distribution of clinically defined OND and AD subjects in the clusters together with the clusters data ranges with respect to the calculated biomarkers cut-off values, we decided to indicate cluster 1 as the “control” cluster and clusters 3–6 as “AD-clusters”. Grouping of the clusters and of the clinical diagnoses showed that cluster 1, the control cluster, was the most distant from the others, followed by cluster 2 which was included in a separated branch with respect to cluster 3–6. The clinical diagnosis showed also a peculiar grouping, with AD, PDD, and DLB in the same branch of the dendrogram, while the other conditions (mostly included in clusters 1 and 2) in a second large branch. CJD clustered separately from all the other conditions being characterized by high Aβ42/Aβ40 ratios and high t-tau values.

According to the prevalence of AD and OND in clusters 1–6, we calculated cut-off values for each biomarker considering AD vs. OND clinical diagnoses, control cluster vs. AD clusters and cluster 2 vs. AD clusters. The results are shown in Table 1.

**TABLE 1:** Cut-off values for the three core AD biomarkers with their 95% CI were calculated by maximizing the Youden’s index for AD vs. OND, between samples belonging to the AD clusters (cluster 3, 4, 5, and 6) and “control” cluster (cluster 1) and between samples belonging to the AD clusters and cluster 2.

	**Aβ42/Aβ40**	**p-tau (pg/ml)**	**t-tau (pg/ml)**
OND vs. AD	0.073 (0.063, 0.079)	53.5 (47.2, 57.5)	371 (332, 393)
Control cluster vs. AD clusters	0.072 (0.070, 0.074)	50.0 (46.2, 52.3)	392 (359, 396)
Cluster 2 vs. AD clusters	0.073 (0.072, 0.078)	71.6 (50.6, 82.8)	1403 (485, 1999)

We noticed that the cut-offs of the comparison control cluster vs. AD clusters were relatively similar to what obtained using the clinical diagnosis grouping (OND vs. AD), being mostly comprised within the 95% CI. Cut-off values for control cluster vs. AD clusters remained unchanged also by considering an age- and gender-matched subsets of the population (Supplementary Figure 2).

On the other hand, the cluster 2 vs. AD clusters comparison showed significant differences in the absolute values of cut-offs for t-tau and p-tau, while the Aβ42/Aβ40 ratio did not change significantly.

## Discussion

In the last decade, CSF Aβ42/Aβ40 ratio, p-tau and t-tau emerged as reliable markers of brain amyloidosis, tauopathy and tau-related neurodegeneration. The introduction of these markers into clinical practice has substantially helped the neurologist to change the definition of AD from a syndromal to a molecular construct (Jack et al., 2018). In particular, considering the well-established A/T/(N) system (Jack et al., 2018), AD is now defined by the presence of both brain amyloidosis (A+) and tauopathy (T+), with neurodegeneration (N+) being a non-necessary condition. The recent advent of automated platforms for core AD biomarker assessment in CSF, has been of substantial help in limiting both intra and inter-assay variability with respect to manual ELISA (Le Bastard et al., 2015). In this work, we used the reproducibility of automated CSF core AD biomarker determination to compare two large cohorts of patients diagnosed with various neurological disorders and enrolled in two centers with proven expertise in AD biomarkers. After a small round-robin validation step on 40 CSF samples, we were able to confirm the good reproducibility of the determinations between the two centers using the automated procedure. In order to overcome the diagnostic heterogeneity of both AD and control groups, we applied unsupervised GMMs to cluster the patients (*n* = 616) according to their CSF biomarker profile and investigate the degree of overlap between the clinical diagnosis and the data-driven classification of the subjects. The data spontaneously grouped in six clusters, 4 of these (clusters 3–6) contained the 95% of clinically defined AD patients (Figure 3), characterized by low Aβ42/Aβ40 values and high values of p-tau and t-tau (Figure 2C). Interestingly, high percentages (>50%) of synucleinopathies with dementia (DLB and PDD) and CBD (33%) fell in these clusters. This fact is not surprising, since the presence of brain amyloidosis and tauopathy is a feature of both DLB and PDD (Irwin et al., 2013; Irwin and Hurtig, 2018; Bellomo et al., 2020b). Cluster 1 instead contained the majority of OND subjects (77%), and PD (84%), MSA (87%), PSP (86%), and MS (100%) patients, suggesting that AD pathology is not frequent in these conditions. This result, together with the high concordance between cut-off values for the OND vs. AD and control cluster vs. AD clusters comparisons, suggest that these conditions may be treated as controls with respect to core AD biomarkers. The inclusion of NPH in control groups should be instead avoided since neurodegeneration and dilution effects may significantly alter the concentration of t-tau and Aβ peptides (Graff-Radford, 2014), in a way that is not fully compensated by computing their ratio. By considering the clusters with highest and lowest frequencies of AD and OND subjects, we were able to compute cluster-defined cut-off values. In our work, the cut-off values calculated by the clustering method were concordant to the ones calculated by relying on clinical diagnoses for AD vs. OND, but this approach may be of more substantial help while facing low numbers of well clinically characterized OND and/or AD subjects. The cut-off values calculated for AD clusters vs. control cluster were substantially unchanged considering age- and gender-matched subsets of the clusters (Supplementary Figure 2), thus reinforcing the reliability of the approach used. Because of these advantages, unsupervised and partially supervised machine-learning algorithms (like the one we applied) have recently started to be applied in neurodegenerative diseases diagnostics (Skillbäck et al., 2015; Racine et al., 2016; Toschi et al., 2019). Moreover, these approaches represent a best choice while dealing with a large number of biomarkers or candidate biomarkers (Solorio-Fernández et al., 2020), e.g., in omics studies (Lopez et al., 2018). The inclusion of a wide panel of CSF markers, possibly linked to different biological pathways, may help in differentiating synucleinopathies with dementia and FTD from AD and synucleinopathies without dementia and PSP from controls. As a limitation of our study, we must report the small sample size of some diagnostic categories (e.g., MS, VAD and PDD). Thus, the frequencies of these categories in clusters may potentially be biased. Another limitation is the lack of amyloid PET, which is of substantial help in identifying brain amyloidosis and well correlates with brain amyloidosis markers such as CSF Aβ42 and Aβ42/Aβ40 (Alcolea et al., 2019; Leitão et al., 2019). However, this partially supervised approach is insensitive to the presence of hidden interfering pathologies in control subjects and to the presence of few AD misdiagnosis, which may occur when the diagnosis is made prevalently by the examination of clinical features. Since the definition of AD and control clusters depended only on the prevalences of clinically defined AD and OND subjects within each cluster, we expect that this approach may provide reliable results as long as the diagnosis/exclusion of AD is correct in the majority of the cases. As an example, considering the biomarker distributions presented in this study, to misclassify the control cluster it would have required at least 28 out of 71 OND subjects (40%) misdiagnosed for AD.

Overall, our findings suggest that automated assays are amenable for large-scale biomarker studies across centers. Furthermore, the use of unsupervised (or partially supervised) machine learning approaches may help the biochemical phenotyping of neurological disorders, being also a robust option for the definition of cut-off values. The implementation of such approaches in biomarker research could substantially improve the development of adequate diagnostic protocols and increase the quality of diagnostic tools for complex and heterogeneous disorders presenting with overlapping clinical syndromes, like dementias.

## Data Availability Statement

Raw data were generated at University of Perugia and Carlo Besta Neurological Institute. Derived data supporting the findings of this study are available from the corresponding authors (LP and GDF) on request.

## Ethics Statement

The studies involving human participants were reviewed and approved by the Ethics Committee from Fondazione IRCCS Istituto Neurologico Carlo Besta, Milan, and University of Perugia. The patients/participants provided their written informed consent to participate in this study.

## Author Contributions

GB analyzed the data and wrote the first draft. AI analyzed the CSF samples at Carlo Besta Neurological Institute and revised the manuscript. DC conceptualized the data analysis and contributed to writing the first draft of the manuscript. EM contributed in analyzing the CSF samples at Carlo Besta Neurological Institute. FPP contributed to the sample collection and assisted with data management of samples. LG contributed to the sample selection and collection. SP and MP analyzed the samples at the University of Perugia and revised the manuscript. FT and GG contributed in the conceptualization of the study and revised the manuscript. GDF and LP designed the study and revised the final version of the manuscript. All the authors read and approved the final version of the manuscript.

## Conflict of Interest

The authors declare that the research was conducted in the absence of any commercial or financial relationships that could be construed as a potential conflict of interest.
