# Supplementary material for: Machine Learning Driven Profiling of Cerebrospinal Fluid Core Biomarkers in Alzheimer’s Disease and Other Neurological Disorders
Source: Front Neurosci. 2021 Mar 31;15:647783. doi: 10.3389/fnins.2021.647783 (PMC8044304; doi:10.3389/fnins.2021.647783)
Supplement: Supplementary file 1 [file Data_Sheet_1.docx]

Supplementary Material

**Machine learning driven profiling of cerebrospinal fluid core biomarkers in Alzheimer’s disease and other neurological disorders**

Giovanni Bellomo^1§^, Antonio Indaco^2§^_,_ Davide Chiasserini^3§^, Emanuela Maderna^2^, Federico Paolini Paoletti^4^, Lorenzo Gaetani^4^, Silvia Paciotti^1^, Maya Petricciuolo^1^, Fabrizio Tagliavini^2^, Giorgio Giaccone^2^, Lucilla Parnetti^1,4^*, Giuseppe Di Fede^2^*

^§^These authors equally contributed to this work and share first authorship

*Corresponding authors; these authors equally contributed to this work and share last authorship

^1^Laboratory of Clinical Neurochemistry, Section of Neurology, Department of Medicine and Surgery, University of Perugia, Perugia, Italy

^2^Neurology 5 / Neuropathology Unit, Fondazione IRCCS Istituto Neurologico C. Besta, Milan, Italy

^3^Section of Biochemistry, Department of Medicine and Surgery, University of Perugia, Perugia, Italy

^4^Section of Neurology, Department of Medicine and Surgery, University of Perugia, Perugia, Italy

***Correspondence:**giuseppe.difede@istituto-besta.it; lucilla.parnetti@unipg.it

**Supplementary Table 1** **|** Diagnostic composition of the validation cohort.

|  | | **Mean** | | | | | **CV** | | | | |
| --- | --- | --- | --- | --- | --- | --- | --- | --- | --- | --- | --- |
| **COHORT** | **DIAGNOSIS** | **Aβ42 (pg/ml)** | **Aβ42/ Aβ40** | **Aβ40 (pg/ml)** | **t-tau (pg/ml)** | **p-tau (pg/ml)** | **Aβ42** | **Aβ42/ Aβ40** | **Aβ40** | **t-tau** | **p-tau** |
| 1 | AD | 408.5 | 0.045 | 9011 | 453 | 71.8 | 4% | 7% | 2% | 11% | 2% |
| 1 | AD | 448.5 | 0.037 | 12076.5 | 794 | 131.1 | 6% | 5% | 0% | 2% | 0% |
| 1 | AD | 662.5 | 0.043 | 15337.5 | 767 | 139.6 | 3% | 4% | 1% | 1% | 1% |
| 1 | AD | 506 | 0.049 | 10248.5 | 445.5 | 73.7 | 3% | 6% | 3% | 2% | 1% |
| 1 | AD | 903.5 | 0.063 | 14490 | 443 | 72.9 | 6% | 8% | 2% | 1% | 1% |
| 1 | AD | 400.5 | 0.035 | 11383 | 771.5 | 144.9 | 5% | 5% | 1% | 2% | 3% |
| 1 | AD | 297 | 0.030 | 9977.5 | 792 | 131.2 | 2% | 5% | 4% | 2% | 4% |
| 1 | AD | 589 | 0.048 | 12301.5 | 1155.5 | 115.1 | 2% | 9% | 7% | 0% | 3% |
| 1 | AD | 538 | 0.070 | 7691 | 608.5 | 93.8 | 3% | 6% | 9% | 17% | 9% |
| 1 | AD | 269 | 0.029 | 9269 | 984.5 | 160.3 | 9% | 5% | 4% | 3% | 1% |
| 1 | AD | 516.5 | 0.057 | 9020.5 | 1267.5 | 177.8 | 9% | 5% | 4% | 2% | 3% |
| 1 | OND | 1122 | 0.096 | 11648 | 356 | 33.9 | 3% | 6% | 3% | 8% | 1% |
| 1 | OND | 1408 | 0.092 | 15302 | 325.5 | 44.3 | 9% | 8% | 0% | 3% | 2% |
| 1 | OND | 1548 | 0.103 | 15048.5 | 341.5 | 46.4 | 9% | 9% | 0% | 4% | 1% |
| 1 | OND | 1128 | 0.105 | 10767.5 | 171 | 24.7 | 11% | 8% | 3% | 4% | 6% |
| 1 | OND | 813 | 0.063 | 12913 | 380 | 56.4 | 7% | 4% | 3% | 4% | 2% |
| 1 | PD | 1070 | 0.087 | 12335.5 | 255.5 | 32.1 | 6% | 6% | 0% | 1% | 0% |
| 1 | PD | 1363.5 | 0.098 | 13939.5 | 215 | 34.0 | 5% | 8% | 3% | 7% | 1% |
| 1 | PD | 1016 | 0.093 | 10896.5 | 197 | 28.5 | 5% | 5% | 0% | 1% | 0% |
| 1 | PD | 1162.5 | 0.096 | 12118.5 | 230.5 | 32.3 | 7% | 5% | 3% | 24% | 2% |
| 2 | AD | 224.5 | 0.061 | 3699 | 376.5 | 55.9 | 21% | 5% | 26% | 6% | 3% |
| 2 | AD | 217 | 0.051 | 4227.5 | 461 | 77.8 | 21% | 1% | 20% | 13% | 6% |
| 2 | AD | 353 | 0.049 | 7242.5 | 366 | 64.7 | 16% | 7% | 8% | 6% | 1% |
| 2 | AD | 418.5 | 0.035 | 12131.5 | 870 | 108.7 | 6% | 12% | 6% | 2% | 4% |
| 2 | AD | 502 | 0.031 | 16118.5 | 888.5 | 168.2 | 22% | 5% | 18% | 22% | 22% |
| 2 | AD | 298.5 | 0.048 | 6203 | 187.5 | 31.5 | 16% | 5% | 12% | 28% | 4% |
| 2 | AD | 339 | 0.053 | 6428 | 417.5 | 73.1 | 8% | 7% | 1% | 8% | 1% |
| 2 | AD | 221.5 | 0.030 | 7429 | 2521.5 | 424.0 | 14% | 8% | 23% | 7% | 5% |
| 2 | AD | 374.5 | 0.036 | 10328 | 550.5 | 85.9 | 24% | 4% | 28% | 10% | 5% |
| 2 | AD | 214.5 | 0.045 | 4784.5 | 1136 | 165.7 | 23% | 9% | 14% | 6% | 8% |
| 2 | DLB | 344.5 | 0.050 | 7082.5 | 413 | 72.2 | 33% | 10% | 42% | 23% | 12% |
| 2 | FTD | 696 | 0.063 | 11041 | 332.5 | 41.7 | 3% | 5% | 8% | 8% | 1% |
| 2 | FTD | 581.5 | 0.091 | 6385.5 | 197.5 | 23.0 | 5% | 5% | 10% | 17% | 12% |
| 2 | MSA-C | 650.5 | 0.103 | 6279.5 | 452 | 39.7 | 13% | 6% | 7% | 8% | 0% |
| 2 | MSA-P | 937.5 | 0.096 | 9735.5 | 293 | 32.0 | 16% | 6% | 10% | 7% | 3% |
| 2 | NPH | 770.5 | 0.106 | 7210.5 | 195 | 27.4 | 28% | 23% | 6% | 15% | 1% |
| 2 | NPH | 666.5 | 0.097 | 6741.5 | 130 | 22.3 | 42% | 19% | 24% | 21% | 6% |
| 2 | PD | 477 | 0.085 | 5571.5 | 236 | 23.8 | 24% | 11% | 13% | 10% | 8% |
| 2 | PSP | 501 | 0.090 | 5557 | 146.5 | 21.1 | 24% | 2% | 22% | 35% | 4% |
| 2 | PSP | 692 | 0.101 | 6860 | 245.5 | 28.4 | 13% | 8% | 5% | 15% | 2% |
|  | | | | | | | | | | | |
|  | | **Aβ42** | | **Aβ42/Aβ40** | | **Aβ40** | **t-tau** | | **p-tau** | | |
| **mean inter-assay CV  (40 samples)** | | 12% | | 7% | | 9% | 9% | | 4% | | |
| **mean intra-assay CV  (QC sample, 3 runs)** | | 3% | | 2% | | 1% | 1% | | 2% | | |

PSP: progressive supranuclear palsy; AD: Alzheimer’s disease; MSA: multiple-system atrophy; FTD: frontotemporal dementia; DLB: dementia with Lewy bodies; PD: Parkinson’s disease; NPH: normal-pressure hydrocephalus; SCD: subjective cognitive decline. Inter-assay coefficients of variation (CV) have been calculated as ratio between standard deviation and mean values obtained on the same samples in the two laboratories. The mean intra-assay coefficient of variation was obtained by performing three consecutive measurements on a quality control sample (QC sample) belonging to a pool of CSF with mean biomarker values of 495 pg/ml Aβ42, 0.068 Aβ42/Aβ40, 7305 pg/ml Aβ40, 387 pg/ml t-tau and 58.7 pg/ml p-tau. The shown mean calculated CV are relative to 3 consecutive biomarker measurements with Lumipulse-G.

**Supplementary Table 2** **|** Results of the Passing-Bablok regression analyses of Aβ40, Aβ42 Aβ42/Aβ40, p-tau and t-tau measured on 40 samples (20 from each cohort) in the two centres.

| **Biomarker** | **Slope** | **Intercept** | **r** |
| --- | --- | --- | --- |
| **Aβ40** | 1.12 (1.07, 1.18) | -1790 (-2280, -1222) pg/ml | 0.95 |
| **Aβ42** | 1.17 (1.06, 1.22) | -97 (-135, -28) pg/ml | 0.95 |
| **Aβ42/Aβ40** | 1.05 (0.89, 1.12) | 0.001 (-0.002, 0.007) | 0.94 |
| **p-tau** | 0.96 (0.93, 1.0) | 1.0 (-0.5, 2.9) pg/ml | 0.99 |
| **t-tau** | 1.0 (0.93, 1.03) | -32 (-50, -8) pg/ml | 0.99 |

Correlations have been calculated in terms of Pearson’s correlation coefficients (r). Fitted slopes and intercepts are reported with their 95% confidence interval (CI) in brackets.

**Supplementary Table 3** **|** Diagnostic groups

| **Acronym** | **Diagnosis** | **N** | **Mean age (y)** | **95% CI Mean (y)** | **Males/Females** |
| --- | --- | --- | --- | --- | --- |
| **AD** | Alzheimer’s disease | 257 | 70.2 | (69.2, 71.1) | 107/150 |
| **OND** | neurological controls | 71 | 63 | (59.6, 66.4) | 34/37 |
| **PD** | Parkinson's disease | 56 | 64.9 | (62.3, 67.4) | 39/17 |
| **FTD** | Frontotemporal dementia | 50 | 67.4 | (65.3, 69.6) | 21/29 |
| **NPH** | Normal-pressure hydrocephalus | 27 | 69.6 | (65.2, 73.9) | 15/12 |
| **MSA** | Multiple system atrophy | 23 | 64 | (60.3, 67.7) | 12/11 |
| **DLB** | Dementia with Lewy bodies | 21 | 70 | (66.5, 73.6) | 16/5 |
| **sMCI** | Stable mild cognitive impairment | 20 | 70.2 | (66.8, 73.5) | 10/10 |
| **PSP** | Progressive supranuclear palsy | 14 | 68.6 | (63.1, 74.0) | 8/6 |
| **CBD** | Cortico-basal degeneration | 12 | 67.7 | (62.8, 72.5) | 5/7 |
| **Park** | Parkinsonism | 9 | 62.3 | (56.6, 68.1) | 4/5 |
| **SCD** | Subjective cognitive decline | 8 | 69 | (63.7, 74.3) | 3/5 |
| **CJD** | Creutzfeldt-Jakob disease | 8 | 66.5 | (57.4, 75.6) | 4/4 |
| **Enc** | Encephalitis | 8 | 63 | (54.0, 72.0) | 5/3 |
| **PDD** | Parkinson's disease with dementia | 7 | 71.4 | (61.4, 81.4) | 6/1 |
| **MS** | Multiple sclerosis | 6 | 59 | (45.9, 72.1) | 2/4 |
| **VaD** | Vascular dementia | 5 | 66.8 | (52.4, 81.1) | 2/2 |
| **CAA** | Cerebral amyloid angiopathy | 3 | 68.3 | (48.1, 88.6) | 3/0 |
| **CVD** | Cerebrovascular disease | 3 | 67 | (40.8, 93.2) | 1/2 |
| **uDEM** | Dementia of unknown origin | 3 | 71.7 | (53.0, 90.3) | 2/1 |
| **SCA** | Spinocerebellar ataxia | 2 | 70 | (55.0, 85.0) | 1/1 |
| **Enclo** | Encephalopathy | 2 | 54.5 | (54.0, 55.0) | 1/1 |
| **Eps** | Epilepsy | 1 | 60 | - | 1/0 |
| **ALS** | Amyotrophic lateral sclerosis | 1 | 69 | - | 0/1 |

Diagnostic groups (together with the acronyms used throughout the manuscript), sample sizes (N), mean age, 95% confidence interval (95%CI) for mean age and the number of males/females are reported in the table for all the 616 subjects.

**Supplementary Table 4** **|** Summary of the Receiver Operating Characteristic (ROC) curve analysis.

| Biomarker | AUC 1+2 | Sensitivity 1+2 | Specificity 1+2 | Cut-off 1+2 | Cut-off 1 | Cut-off 2 |
| --- | --- | --- | --- | --- | --- | --- |
| Aβ42 | 0.93  (0.90, 0.97) | 0.92  (0.82, 0.97) | 0.86  (0.81, 0.93) | 636  (598, 742) pg/ml | 735  (596, 796) pg/ml | 617  (511, 653) pg/ml |
| Aβ42/Aβ40 | 0.97  (0.95, 0.99) | 0.94  (0.89, 0.99) | 0.96  (0.91, 0.98) | 0.073  (0.063, 0.079) | 0.069  (0.062, 0.073) | 0.074  (0.072, 0.087) |
| p-tau | 0.96  (0.94, 0.98) | 0.93  (0.86, 0.99) | 0.91  (0.86, 0.96) | 53.5  (47.2, 57.5) pg/ml | 57.4  (47.2, 59.8) pg/ml | 50.2  (42.0, 54.0) pg/ml |
| t-tau | 0.91  (0.86, 0.95) | 0.87  (0.79, 0.94) | 0.91  (0.87, 0.95) | 371  (338, 392) pg/ml | 395  (341, 463) pg/ml | 353  (274, 361) pg/ml |

95%CI were calculated with the bootstrap method. Cut-off values for the three core AD biomarkers with their 95% CI were calculated by maximizing the Youden’s index for AD (N = 257) vs OND (N = 71)of the two merged cohorts (1+2), cohort 1 (University of Perugia, N AD = 137 AD, N OND = 40) and cohort 2 (Carlo Besta Neurological Institute, N AD = 120, N OND = 31). For the comparisons cluster 1+cluster 2 (1+2), areas under the ROC curves (AUC), sensitivity and specificity are also reported.


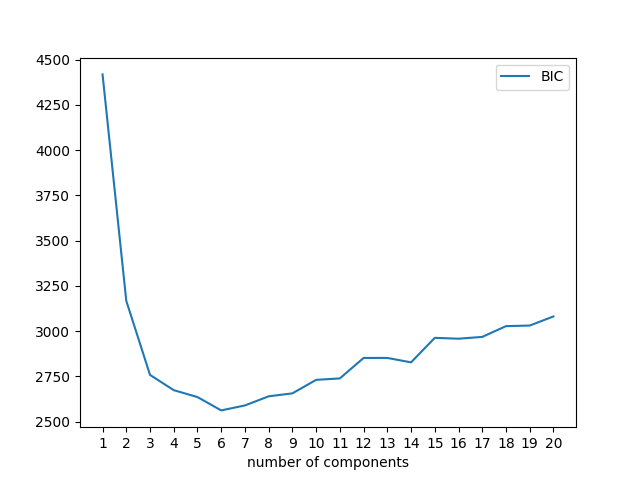


Supplementary Figure 1 | Bayesian information criterion (BIC) function vs number of clusters used for the Gaussian-Mixture Model (GMM) clustering. The BIC minimum was reached by imposing a number of clusters equal to 6.

$f_{\boldsymbol{x}}\left( x_{1},\ldots, x_{k} \right)=\frac{\exp\left( -\frac{1}{2}\left( \boldsymbol{x}-\boldsymbol{\mu} \right)^{T}\Sigma^{-1}\left( \boldsymbol{x}-\boldsymbol{\mu} \right) \right)}{\sqrt{\left( 2\pi\right)^{k}\left| \Sigma\right|}}$ (S1)

**Supplementary Table 5** **|** With reference to Eq. S1.

| z-space | | | |  | biomarker space | | |
| --- | --- | --- | --- | --- | --- | --- | --- |
| Centroids | | | | | | | |
|  | Aβ42/Aβ40 | p-tau | t-tau |  | Aβ42/Aβ40 | p-tau (pg/ml) | t-tau (pg/ml) |
| μ_1_ | 0.739761 | -0.70087 | -0.68768 |  | 0.098025 | 30.64856 | 250.2223 |
| μ_2_ | 1.100505 | -0.26499 | 0.781868 |  | 0.109385 | 56.36583 | 878.017 |
| μ_3_ | -0.68401 | -0.13575 | -0.27549 |  | 0.05319 | 63.99047 | 426.3117 |
| μ_4_ | -1.03342 | 0.727053 | 0.35536 |  | 0.042187 | 114.8961 | 695.8112 |
| μ_5_ | -0.89863 | 1.270915 | 1.079675 |  | 0.046432 | 146.984 | 1005.241 |
| μ_6_ | -1.13867 | 2.953557 | 2.685178 |  | 0.038873 | 246.2598 | 1691.118 |
| Covariance matrices | | | | | | | |
| Σ_1_ | 0.277951 | -0.00452 | 0.001044 |  | 0.000276 | -0.0084 | 0.014047 |
|  | 0.001044 | 0.020277 | 0.035877 |  | 0.014047 | 511.0819 | 6547.742 |
|  | -0.00452 | 0.023748 | 0.020277 |  | -0.0084 | 82.66553 | 511.0819 |
|  |  |  |  |  |  |  |  |
| Σ_2_ | 0.984267 | -0.16364 | -0.60985 |  | 0.000976 | -0.30402 | -8.20415 |
|  | -0.60985 | 0.302747 | 2.396454 |  | -8.20415 | 7630.752 | 437360.4 |
|  | -0.16364 | 0.389759 | 0.302747 |  | -0.30402 | 1356.751 | 7630.752 |
|  |  |  |  |  |  |  |  |
| Σ_3_ | 0.144216 | -0.02914 | -0.01239 |  | 0.000143 | -0.05414 | -0.16664 |
|  | -0.01239 | 0.085113 | 0.085784 |  | -0.16664 | 2145.284 | 15655.78 |
|  | -0.02914 | 0.102271 | 0.085113 |  | -0.05414 | 356.0041 | 2145.284 |
|  |  |  |  |  |  |  |  |
| Σ_4_ | 0.053441 | -0.02284 | -0.02537 |  | 5.3E-05 | -0.04243 | -0.34125 |
|  | -0.02537 | 0.156786 | 0.142083 |  | -0.34125 | 3951.79 | 25930.68 |
|  | -0.02284 | 0.235283 | 0.156786 |  | -0.04243 | 819.0192 | 3951.79 |
|  |  |  |  |  |  |  |  |
| Σ_5_ | 0.116848 | -0.065 | -0.05422 |  | 0.000116 | -0.12077 | -0.72937 |
|  | -0.05422 | 0.270969 | 0.21355 |  | -0.72937 | 6829.771 | 38973.5 |
|  | -0.065 | 0.541028 | 0.270969 |  | -0.12077 | 1883.32 | 6829.771 |
|  |  |  |  |  |  |  |  |
| Σ_6_ | 0.044246 | -0.24188 | -0.11332 |  | 4.39E-05 | -0.4494 | -1.52447 |
|  | -0.11332 | 1.136852 | 0.564009 |  | -1.52447 | 28654.39 | 102933.5 |
|  | -0.24188 | 2.470915 | 1.136852 |  | -0.4494 | 8601.254 | 28654.39 |

We report the fitted GMM parameters, centroids (μ_k_) and covariance matrices (Σ_k_) both in the Z-space and in the original Biomarkers space.


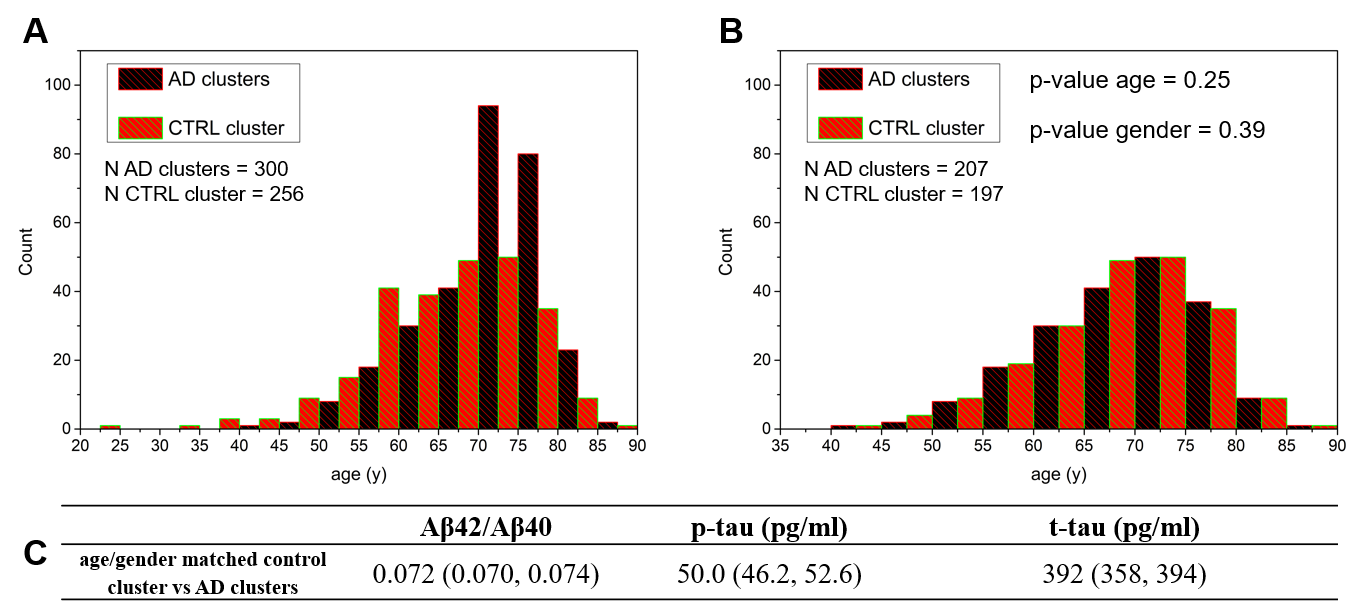


Supplementary Figure 2 Age histograms of the original study population in AD and control clusters (A) and of the age/sex-matched subsets (B). Age histogram matching was performed by random exclusion of subjects within bins of 5 years width. Exclusion of samples according to gender was subsequently conducted until p-values > 0.25 were obtained by logistic regression both for age and gender. C) Recalculated cutoff values for the age- and gender-matched subset for control cluster vs AD clusters.
